# Multiparametric imaging of patient and tumour heterogeneity in non-small-cell lung cancer: quantification of tumour hypoxia, metabolism and perfusion

**DOI:** 10.1007/s00259-015-3169-4

**Published:** 2015-09-04

**Authors:** Wouter van Elmpt, Catharina M. L. Zegers, Bart Reymen, Aniek J. G. Even, Anne-Marie C. Dingemans, Michel Oellers, Joachim E. Wildberger, Felix M. Mottaghy, Marco Das, Esther G. C. Troost, Philippe Lambin

**Affiliations:** Department of Radiation Oncology (MAASTRO), GROW – School for Oncology and Developmental Biology, Maastricht University Medical Centre, Maastricht, The Netherlands; Department of Pulmonology, GROW – School for Oncology and Developmental Biology, Maastricht University Medical Centre, Maastricht, The Netherlands; Department of Radiology, GROW – School for Oncology and Developmental Biology, Maastricht University Medical Centre, Maastricht, The Netherlands; Department of Nuclear Medicine, GROW – School for Oncology and Developmental Biology, Maastricht University Medical Centre, Maastricht, The Netherlands; Department of Nuclear Medicine, University Hospital RWTH Aachen University, Aachen, Germany

**Keywords:** DCE CT, Hypoxia PET, HX4, FDG PET, Image analysis, Multiparametric

## Abstract

**Purpose:**

Multiple imaging techniques are nowadays available for clinical in-vivo visualization of tumour biology. FDG PET/CT identifies increased tumour metabolism, hypoxia PET visualizes tumour oxygenation and dynamic contrast-enhanced (DCE) CT characterizes vasculature and morphology. We explored the relationships among these biological features in patients with non-small-cell lung cancer (NSCLC) at both the patient level and the tumour subvolume level.

**Methods:**

A group of 14 NSCLC patients from two ongoing clinical trials (NCT01024829 and NCT01210378) were scanned using FDG PET/CT, HX4 PET/CT and DCE CT prior to chemoradiotherapy. Standardized uptake values (SUV) in the primary tumour were calculated for the FDG and hypoxia HX4 PET/CT scans. For hypoxia imaging, the hypoxic volume, fraction and tumour-to-blood ratio (TBR) were also defined. Blood flow and blood volume were obtained from DCE CT imaging. A tumour subvolume analysis was used to quantify the spatial overlap between subvolumes.

**Results:**

At the patient level, negative correlations were observed between blood flow and the hypoxia parameters (TBR >1.2): hypoxic volume (−0.65, *p* = 0.014), hypoxic fraction (−0.60, *p* = 0.025) and TBR (−0.56, *p* = 0.042). At the tumour subvolume level, hypoxic and metabolically active subvolumes showed an overlap of 53 ± 36 %. Overlap between hypoxic sub-volumes and those with high blood flow and blood volume was smaller: 15 ± 17 % and 28 ± 28 %, respectively. Half of the patients showed a spatial mismatch (overlap <5 %) between increased blood flow and hypoxia.

**Conclusion:**

The biological imaging features defined in NSCLC tumours showed large interpatient and intratumour variability. There was overlap between hypoxic and metabolically active subvolumes in the majority of tumours, there was spatial mismatch between regions with high blood flow and those with increased hypoxia.

**Electronic supplementary material:**

The online version of this article (doi:10.1007/s00259-015-3169-4) contains supplementary material, which is available to authorized users.

## Introduction

Locally advanced stage non-small-cell (NSCLC) lung cancer still has a poor prognosis, despite more advanced and aggressive treatment strategies consisting of a combination of chemotherapy and radiotherapy. Integrated ^18^F-FDG PET/CT of NSCLC is recommended for assessment of the primary tumour extension, and detection of regional lymph node metastases and possibly distant metastases. Tumour heterogeneity assessed by noninvasive imaging might be helpful in guiding treatment planning and optimizing outcome [1–6]. High levels of hypoxia and metabolic activity are linked to a poor prognosis. Furthermore, as known from histology, vascular parameters such as blood flow and blood volume are decreased in hypoxic tumours [7].

Several imaging modalities are currently available for identifying different, biologically heterogeneous regions. Tumour metabolism can be imaged using FDG PET and is a known prognostic marker for outcome. Response assessment with repeated FDG PET/CT has been shown to have predictive power in NSCLC [8, 9]. Hypoxia is another well-known characteristic of solid tumours that has negative effects on sensitivity to radiotherapy and chemotherapy, while increasing the metastatic potential of cancer cells. Imaging of hypoxia is currently possible using nitroimidazole-based PET tracers such as FMISO or HX4 [10–12]. Perfusion of tumours is important in supplying the tumour with nutrients and oxygen for expansive growth. In addition, targeted agents have been developed that influence perfusion of the tumour by inhibition of angioneogenesis [13]. Imaging of vascular properties of tumours is technically feasible using dynamic contrast-enhanced (DCE) CT or DCE MRI that allow the characterization of various vascular parameters such as blood flow and permeability [14, 15].

The clinical benefit of multiparametric imaging may allow comprehensive assessment of the biological status of the tumour that can subsequently be used for individualized therapy. Especially with newly developed chemotherapy and agents targeting specific biological characteristics, such as hypoxia within the tumour, that are currently under investigation, the use of molecular imaging for treatment selection and response assessment has become important [16]. Furthermore, using modern radiotherapy techniques it is possible to modulate the radiation dose in the tumour volume to boost subvolumes that are likely to be more therapy-resistant [17–20].

Therefore, in this study we investigated multiple imaging techniques to assess the metabolic, hypoxic and vascular status of the primary tumour in a cohort of patients with advanced stage NSCLC. We performed an analysis at the patient level to compare the various biological characteristics in the primary tumours. Next, we investigated possible correlations at the subvolume level of the primary tumour for each individual patient to identify spatial agreement (or mismatch) at the tumour subvolume level.

## Materials and methods

Patients with stage II–IV NSCLC were included from two ongoing therapeutic clinical trials (NCT01024829 and NCT01210378) that have the same pretreatment imaging procedures. Apart from the standard FDG PET/CT imaging for radiation treatment planning, patients received an additional DCE CT scan and a hypoxia HX4 PET/CT scan. Preferably, the DCE CT and HX4 PET/CT scans were acquired on the same day. The FDG PET/CT scan for treatment planning purposes was obtained in the same week and typically prior to the other scans. The study was approved by the appropriate medical ethics committee, and written and signed informed consent was obtained from all individual participants included in the studies.

### FDG PET/CT acquisition

The FDG PET/CT scan was acquired on a Biograph 40 PET/CT scanner (Siemens Healthcare, Forchheim, Germany) 1 h after injection of approximately 250 MBq FDG. Reconstruction settings for the PET scan were based on an iterative reconstruction algorithm with attenuation and scatter correction (OSEM, four iterations, eight subsets, using a 5-mm Gaussian filter) on a 168 × 168 (4 × 4 mm) grid and a slice thickness of 3 mm . The FDG PET/CT imaging procedure was accredited following EANM/EARL guidelines [21]. A respiration-correlated (4D) CT scan was acquired on the PET/CT scanner with the midventilation phase of the CT scan used for PET attenuation correction. The midventilation phase was fused with the FDG PET images and used for contouring the gross tumour volume necessary for radiation treatment planning.

### Dynamic contrast-enhanced CT acquisition

The DCE CT acquisition was performed on a second-generation dual source CT scanner (SOMATOM Definition Flash; Siemens Healthcare) using a previously described acquisition protocol [22]. In brief, the CT scan comprised a first-pass volumetric perfusion acquisition with a serial acquisition every 1.5 s at 80 kVp CT of the part of the thorax centred on the primary tumour (scan length 12 cm) for a period of approximately 50 s (33 consecutive scans). Patients were imaged in expiration breath-hold and asked to continue shallow breathing if holding their breath was no longer possible. Patients received 60 ml of iodinated contrast medium (Iopromide 300; Bayer Healthcare, Berlin, Germany) at a flow rate of 7 ml/s (iodine delivery rate 2.1 g/s), followed by a saline chaser of 30 ml at the same flow rate. CT images were reconstructed using a B20f filter with a slice thickness of 5 mm and a slice increment of 3 mm. CT images were registered afterwards to correct for breathing motion and a deconvolution approach was used for the kinetic modelling of the perfusion parameters implemented in commercially available software (Siemens VPCT body; Siemens Healthcare). The perfusion parameters blood flow (ml/100 ml/min) and blood volume (ml/100 ml) were extracted. A detailed description of the imaging and analysis techniques for DCE CT imaging has recently been described [23, 24].

### Hypoxia HX4 PET/CT scan

Hypoxia PET imaging was performed using the hypoxia PET tracer HX4 (Threshold Pharmaceuticals). The characteristics of this tracer and acquisition details have been described elsewhere [25]. A dose of approximately 440 MBq of HX4 was administered intravenously. The scan acquired at 4 h after injection was used for analysis. Images were acquired using a time-of-flight PET/CT scanner (Gemini TF64; Philips, The Netherlands) with reconstructed images (BLOB-OS-TF, three iterations, 33 subsets) having an in-plane pixel spacing of 4 mm at a slice thickness of 4 mm without overlap.

### Image preprocessing

For the FDG PET and HX4 PET scans, the image intensities were converted to standardized uptake values (SUV) correcting for decay, injected dose and body weight. Additionally for the hypoxia PET scans, a region of interest in the aorta was drawn to normalize tumour uptake to the average background uptake in the blood pool. A tumour-to-blood ratio (TBR) was calculated for every voxel. The hypoxic fraction and volume were then calculated using a TBR >1.2 and TBR >1.4 within the volume of the primary tumour. Next all CT scans were nonrigidly registered to the CT scan of the FDG PET/CT scan using a previously validated deformable registration algorithm [26–28]. The hypoxia PET image and DCE CT extracted kinetic parameter maps were registered using the CT-derived deformation field for that dataset. All deformation fields were visually inspected and approved.

### Multiparametric image analysis

In total four parameter maps, including the metabolic FDG PET scan, the hypoxic HX4 PET scan and two parameters derived from the DCE CT imaging (blood flow and blood volume), were investigated for correlations. Analysis was performed at two levels: correlation at the patient level and investigation at the tumour subvolume level. First, at the patient level we investigated the average uptake or signal of the four parameter maps at the global tumour level. For this we calculated the average of the parameter maps within the primary tumour, i.e. mean SUV of FDG uptake, average TBR, hypoxic volume and hypoxic fraction of the hypoxia scan and average blood flow and volume for the DCE CT kinetic analysis. Second, we analysed subvolumes within the primary tumour by selecting the regions with elevated characteristics as frequently used in previous studies. For the FDG PET scan we used the area with an SUV larger than 50 % of the maximum SUV: i.e. the high FDG uptake volume [17, 29]. Hypoxic regions of the HX4 PET scans were segmented according to threshold values with TBRs larger than 1.2 and 1.4 [25]. For DCE CT imaging, clear thresholds have not been defined. We therefore chose the regions inside the tumour that were more than 50 % of the upper first percentile. We did not choose the single voxel with maximum blood flow or volume as the histograms turned out to be skewed towards high values possibly also with noise that could have influenced the maximum value. The overlaps between the various subvolumes were calculated. Since tumour volumes have a large range between patients, we normalized all values obtained to the volume of high FDG uptake in the individual patient.

### Statistics

Correlation coefficients were calculated using Spearman’s correlation coefficient (MATLAB and Statistics Toolbox Release 2012b; The MathWorks, Inc., Natick, MA). Differences among subgroups of the population were tested using the Wilcoxon signed ranks test. Overlapping subvolumes are presented as Venn diagrams and values were normalized to the volume defined by the high FDG uptake. *P* values <0.05 were assumed to be statistically significant and averages are presented as means ± standard deviation (SD).

## Results

**Fig. 1:**
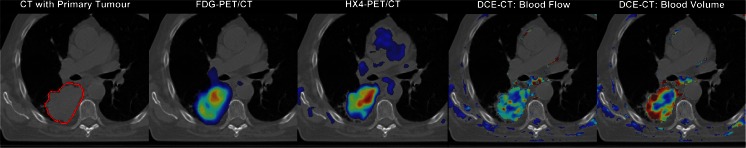
Example of multiparametric imaging in a patient with NSCLC in the right lower lobe. *Left to right*: CT image with the primary tumour delineated (in *red*), metabolic activity imaged with FDG PET/CT, hypoxia imaged with HX4 PET/CT, and perfusion parameters (blood flow and blood volume) depicted with DCE CT

Between March 2012 and March 2014, 21 patients from the two clinical trials mentioned above who underwent imaging with all three modalities were included in this analysis. Patients were excluded from this analysis for various reasons: tumour located at the diaphragm with a large motion vector (>1 cm) as assessed on the 4D CT scan (one patient); acquisition or calculation problems, e.g. too long or short delay between injection and start of the DCE CT scan (three patients) or a calculation error in the vascular or deformation maps (two patients); and an FDG PET scan that was not acquired 1 h after injection (one patient). In total 14 patients were identified who successfully underwent all three imaging modalities, having a median of 2 days between all scans (range 1–6 days). The characteristics of the patients are shown in Table 1. An example of the various images in a patient with a tumour in the right lower lobe is shown in Fig. 1.

**Table 1:** Characteristics of the 14 included patients

Characteristic	Value
Gender, *n* (%)	
Male	11 (79)
Female	3 (21)
Stage, *n* (%)	
IIBp	1 (7)
IIIA	6 (43)
IIIB	6 (43)
IV^a^	1 (7)
Gross tumour volume (cm^3^)	
Mean ± SD (median)	149 ± 200 (86.4)
Range	10.0 – 784
Pathology, *n* (%)	
Adenocarcinoma	8 (57)
Squamous cell carcinoma	4 (29)
Large-cell carcinoma	2 (14)

^a^Oligometastatic brain metastasis, treated with curative intent

### Population averages and correlations

**Fig. 2:**
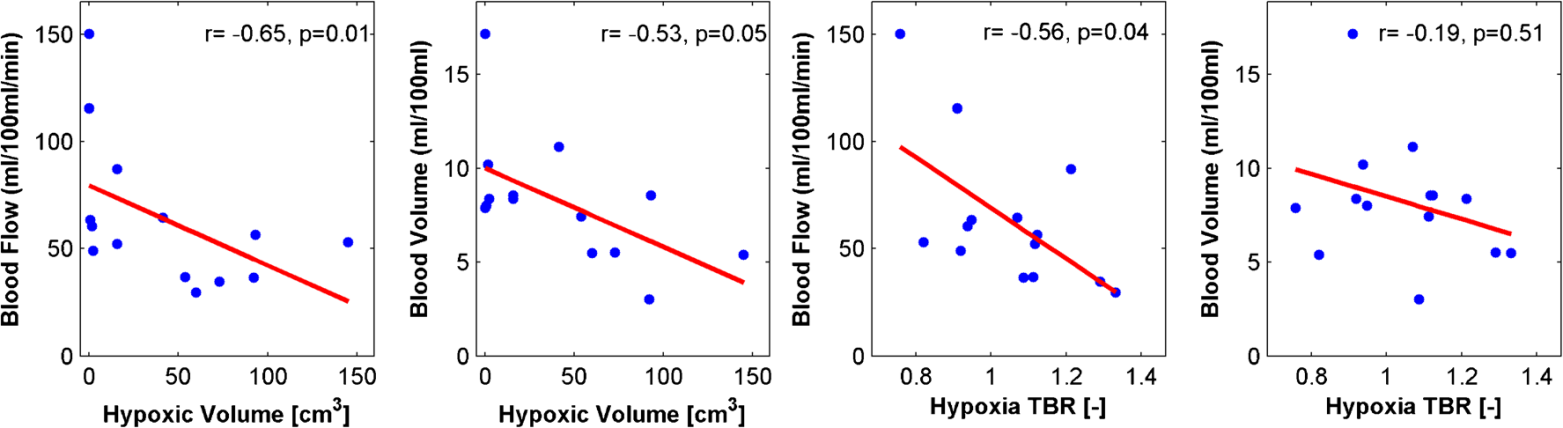
Relationships between hypoxic volumes (*left two plots*) and average hypoxic TBR (*right two plots*) and averaged perfusion parameters blood flow and blood volume in the primary tumour in each patient. Hypoxic volume is defined as the volume within the primary tumour with a TBR >1.2. Tumours with larger hypoxic volumes or increased TBR levels have impaired blood flow

In every patient the average of the parameter of interest of each imaging modality in the primary tumour was calculated. Correlations between averaged perfusion and metabolic parameters, and the hypoxic fraction and hypoxic volume are shown in Fig. 2. There were significant negative correlations between perfusion and hypoxic parameters. There was a negative correlation between hypoxia TBR averaged in the primary tumour and blood flow (−0.56, *p* = 0.042). There were negative correlations between hypoxic volumes defined using TBR thresholds of 1.2 and 1.4 and blood flow (−0.65, *p* = 0.014, and −0.68, *p* = 0.01 respectively). There were also significant negative correlations between hypoxia defined using TBR thresholds of 1.2 and 1.4 and blood volume (−0.60, *p* = 0.025, and −0.62, *p* = 0.02, respectively).

 Correlation coefficients between parameters from the different modalities are shown in Table 2. There were significant correlations within each imaging modality, e.g. between hypoxic fraction and volume for a TBR >1.2 (*r* = 0.657, *p* = 0.013), between hypoxic fraction and TBR (*r* = 0.938, *p* < 0.001), and between blood flow and blood volume (*r* = 0.591, *p* = 0.029). There were no significant correlations between the average FDG SUV and the hypoxic or perfusion parameters. There were no significant correlations between maximum values of the parameters of interest from each imaging modality except hypoxic volume (*R* = 0.85, *p* < 0.001) and hypoxic fraction (*R* = 0.82, *p* < 0.001) defined at a TBR >1.2, both of which were correlated with the maximum FDG SUV. Typically, the maxima were at spatially distinct locations in the tumour volume for different modalities.

**Table 2:** Correlations (Spearman’s correlation coefficients) among the parameters from the different imaging modalities at the population level (14 patients)

		Hypoxia PET			FDG PET	DCE CT	
		Hypoxic volume	Hypoxic fraction	Hypoxia (TBR)	Mean SUV	Blood flow	Blood volume
Hypoxia PET	Hypoxic volume (TBR >1.2)		*p* = **0.013**	*p* = 0.094	*p* = 0.704	*p* = **0.014**	0.052
	Hypoxic fraction (TBR >1.2)	*r* = **0.657**		*p* **< 0.001**	*p* = 0.773	*p* = **0.025**	*p* = 0.175
	Hypoxia (TBR)	*r* = 0.468	*r* = **0.938**		*p* = 0.584	*p* = **0.042**	*p* = 0.512
FDG PET	Mean SUV	*r* = 0.112	*r* = 0.086	*r* = 0.160		*p* = 0.682	*p* = 0.671
DCE CT	Blood flow	*r* = **−0.653**	*r* = **−0.604**	*r* = **−0.556**	*r* = −0.121		*p* = **0.029**
	Blood volume	*r* = −0.534	*r* = −0.385	*r* = −0.191	*r* = −0.125	*r* = **0.591**	

Significant values are indicated in bold

For calculating the correlation coefficients, the various individual parameters (except for hypoxic volume and fraction) were averaged over the primary tumour and represented the average value of the entire volume.

See Supplementary Table 1 for correlation coefficients and *p* values for the hypoxia threshold TBR >1.4

### Subvolume-based analysis

**Fig. 3:**
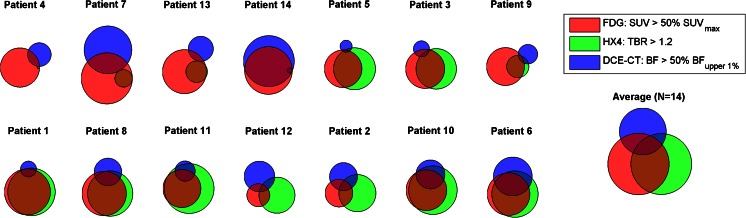
Venn diagrams showing schematically the overlap between the high metabolic regions (FDG, *red*), hypoxic regions with a TBR of >1.2 (HX4, *green*) and increased perfusion blood flow regions (DCE CT, *blue*) in each patient. The patients are ordered according to the amount of overlap between hypoxia and blood flow. The diagram on the *right* shows the average overlap volumes for all patients. Venn diagrams for hypoxic regions defined using a TBR of >1.4 are shown in Supplementary Fig. 1

The average high FDG uptake subvolume was 27.6 ± 24.3 cm^3^ (range 1.6 – 74.7 cm^3^) which was on average 24 ± 10 % of the primary tumour. The average hypoxic subvolume defined by the TBR threshold >1.2 (normalized to the high FDG uptake subvolume of the same patient) was approximately equal to the high FDG uptake volume (98 ± 75 %), and the hypoxic subvolume defined by the TBR threshold >1.4 was 38 ± 43 %. The average normalized volume of high blood flow and high blood volume were 58 ± 48 % and 110 ± 99 %, respectively, again normalized to the high FDG uptake subvolume. There was a large variation in size of the subvolumes. The variation between the various subvolumes and their overlap is visualized in Fig. 3 (numerical data in Table 3). Large but not complete overlap between the hypoxic and high metabolic subvolumes was present. On average in all patients the overlap of the high metabolic volume was 53 ± 36 % (72 ± 27 % expressed relative to the hypoxic volume) for TBR >1.2. In patients with a hypoxic volume defined using TBR >1.4 (nine patients) the overlap of the hypoxic volume was 71 ± 26 %. However, the overlaps between metabolic and hypoxic subvolumes in the high blood flow regions of the tumour were significantly smaller (21 ± 21 % for TBR >1.4 and 15 ± 17 % for TBR >1.2). The overlap between metabolic volume

**Table 3:** Overview of the average tumour subvolumes of the imaging parameters of interest, together with overlap percentages

Patient no.	Volume relative to high FDG volume (%)				Overlap high FDG volume (%) with				Overlap percentage hypoxic volume with			
	Hypoxia		Blood flow	Blood volume	Hypoxia		Blood flow	Blood volume	TBR >1.2		TBR >1.4	
	TBR >1.2	TBR >1.4			TBR >1.2	TBR >1.4			Blood flow	Blood volume	Blood flow	Blood volume
1	107	30	12	111	92	30	6	56	6	61	3	8
2	181	55	102	190	42	9	27	50	30	63	7	13
3	107	43	17	44	67	31	7	26	2	21	0	12
4	0	0	36	175	0	0	9	67	0	0	0	0
5	125	28	10	12	71	23	1	1	2	2	0	0
6	119	46	83	79	89	40	41	38	48	44	17	14
7	12	0	87	95	12	0	29	47	0	1	0	0
8	104	59	38	36	85	55	17	13	18	17	3	5
9	35	0	26	107	28	0	2	15	4	2	0	0
10	143	98	51	77	95	76	32	54	40	63	27	41
11	172	147	28	60	98	88	15	34	25	56	18	47
12	247	24	178	407	40	11	25	64	27	54	2	3
13	24	0	32	36	23	0	5	3	0	0	0	0
14	1	0	114	106	1	0	81	76	1	1	0	0
Average ± SD	98 ± 75	38 ± 43	58 ± 48	110 ± 99	53 ± 36	26 ± 30	21 ± 21	39 ± 24	15 ± 17	28 ± 28	6 ± 9	4 ± 15
Median (range)	107 (0–247)	29 (0–147)	37 (10–178)	87 (12–407)	55 (0–98)	17 (0–88)	16 (1–81)	42 (1–76)	5 (0–48)	19 (0–63)	1 (0–27)	4 (0–47)

Results are normalized to the high FDG uptake volume (i.e. volume with an SUV >50 % of maximum SUV). The high hypoxic volume was defined as the volume with a TBR >1.2 or TBR >1.4; increased blood flow and blood volume levels were defined as the volume with a flow or volume >50 % of the upper first percentile

## Discussion

To our knowledge this is the first study to investigate the relationships among hypoxia, metabolism and perfusion in primary NSCLC tumours at the patient population level and also identified (non)overlapping subvolumes within the tumour.

At the population level, for the average extracted parameters of the entire primary tumour, there was a negative correlation (−0.5 to −0.6) between hypoxia-related parameters (TBR, hypoxic fraction and hypoxic volume) and the perfusion parameter blood flow. As shown in Fig. 2, well-perfused tumours typically showed reduced hypoxia levels. Increased levels of hypoxia are known from multiple studies to have a negative effect on prognosis, and perfusion-limited hypoxia could potentially be detected using an easily available DCE CT imaging protocol. Also, tumours with low perfusion have been reported to have increased potential for lymph node metastases in advanced lung cancer [30]. However, in head-and-neck cancer some investigators have reported that increased blood flow levels are correlated with worse local control [31, 32]. In lung cancer, there is no strong evidence yet that perfusion CT has a large impact on staging for diagnosis [30, 33] or survival prediction [34], but large trials investigating outcome are still lacking. Furthermore, no consensus exists on the optimal CT perfusion protocol, e.g. acquisition (timing) protocol and contrast medium administration, making comparison between different studies difficult [35, 36].

In general, single modality investigations have focused on one biological characteristic of the tumour only. Multimodality imaging might provide more insight into the tumour biology and allow detailed assessment of correlations among tumour characteristics [37–39]. In a recent study in a group of NSCLC patients, we showed a good correlation between hypoxic and metabolically active subvolumes in the majority of the patients [40]. However, there were also patients with no correlation at all, and patients with a partial mismatch between high glucose metabolism and hypoxic regions. Regarding the relationship between perfusion and glucose metabolism, we have previously investigated the correlation between perfusion values within the highly metabolic regions identified with an SUV larger than 50 % of the maximum SUV [22]. We were not able to detect any differences between high and low/moderate FDG uptake regions. This also holds true for the current analysis at the subvolume level and is consistent with observations reported in the literature [30, 41, 42].

Differences have been reported between the NSCLC subtypes adenocarcinoma and squamous cell carcinoma regarding perfusion related parameters [33] and staining of markers of hypoxia and metabolism (e.g. CAIX, GLUT-1) in pathological tumour specimens [43]. We did not find a correlation in the subgroups among the different histological types (data not shown) and speculate that in this study such an analysis may have been hampered by the small size of the subgroups.

In subvolume analysis, registration of the different datasets is of the utmost importance. To minimize gross registration problems we imaged all patients with the same dedicated radiotherapy fixation and support systems including a flat table top on the scanners with dedicated knee roll and arm support systems. Taking into account that imaging was typically performed within the same week, we would not have expected large differences in patient and tumour morphology. Artefacts due to breathing were investigated using the 4D CT scan obtained during FDG PET/CT imaging for radiotherapy purposes, and patients with large tumour motion were excluded from further analysis. Residual uncertainties and small anatomical differences were finally corrected using nonrigid registration that was previously validated [26–28]. Subvolumes in this study were defined by thresholding and the subsequent analysis used the averages within these subvolumes. This approach is quite robust against small errors and noise in the image acquisition and processing for defining the boundary of the segmentation (i.e. the edge of the subvolume). The cut-off values applied have the greatest influence on the definition of the subvolumes, but there is no consensus as to which values to use. For example for hypoxia, several groups have used a TBR cut-off value of >1.4 which implies an increase in the background (blood pool or muscle) needs to be more than 40 % to be classified as hypoxic [40, 44–46]. Other investigators have chosen a TBR >1.2 [47, 48] or even a TBR >1.0 [49]. To our knowledge there is no current standard yet for the definition of a hypoxic volume.

In this study we deliberately did not perform a voxel-by-voxel analysis, because of the need for a high-precision registration technique to match the various imaging modalities. It has been reported that misregistration of even a single voxel can reduce correlation coefficients by 30 % [39]. Furthermore, all the statistical analysis techniques currently applied for finding correlations depend on the assumption that voxels are independent samples, whereas all the underlying imaging techniques exhibit some sort of dependence on neighbouring voxels due to limited resolution (e.g. FWHM of the PET scanner), also calculation of *p* values for assessment of 10,000+ voxels will result in statistical significant correlations for clinically insignificant very small non-zero correlation coefficients.

In the subvolume analysis, there was a large variation in overlap between different patients and tumours. In agreement with literature [40], there was in general overlap between metabolically active and hypoxic volumes, whereas subvolumes with higher blood flow did not show overlap with either metabolically active or hypoxic regions in the majority of the investigated patients. Findings at both the population and subvolume levels support the hypothesis that perfusion-limited hypoxia is related to the vasculature of the tumour [50].

In summary, at the population level we observed an inverse correlation between perfusion-derived parameters, e.g. blood flow and blood volume, and the severity of hypoxia as indicated by the TBR or hypoxic volume. At the tumour subvolume level, correlations between hypoxia and metabolic parameters of perfusion were more variable with the majority of patients showing a spatial mismatch between highly perfused regions and hypoxic volumes within the primary tumour. In future a detailed assessment should be performed to identify subpopulations and subvolumes that might have value for predicting treatment outcomes (e.g. local recurrence) and might thus be used in treatment adaptation (i.e. dose escalation).

## Electronic supplementary material

Suppl. Fig. 1 (DOCX 77 kb)

Suppl. Table 1 (DOCX 22 kb)
