# Supplementary material for: Multiparametric imaging of patient and tumour heterogeneity in non-small-cell lung cancer: quantification of tumour hypoxia, metabolism and perfusion
Source: Eur J Nucl Med Mol Imaging. 2015 Sep 4;43:240–8. doi: 10.1007/s00259-015-3169-4 (PMC4700090; doi:10.1007/s00259-015-3169-4)
Supplement: Supplementary file 1 — (DOCX 77 kb) [file 259_2015_3169_MOESM1_ESM.docx]

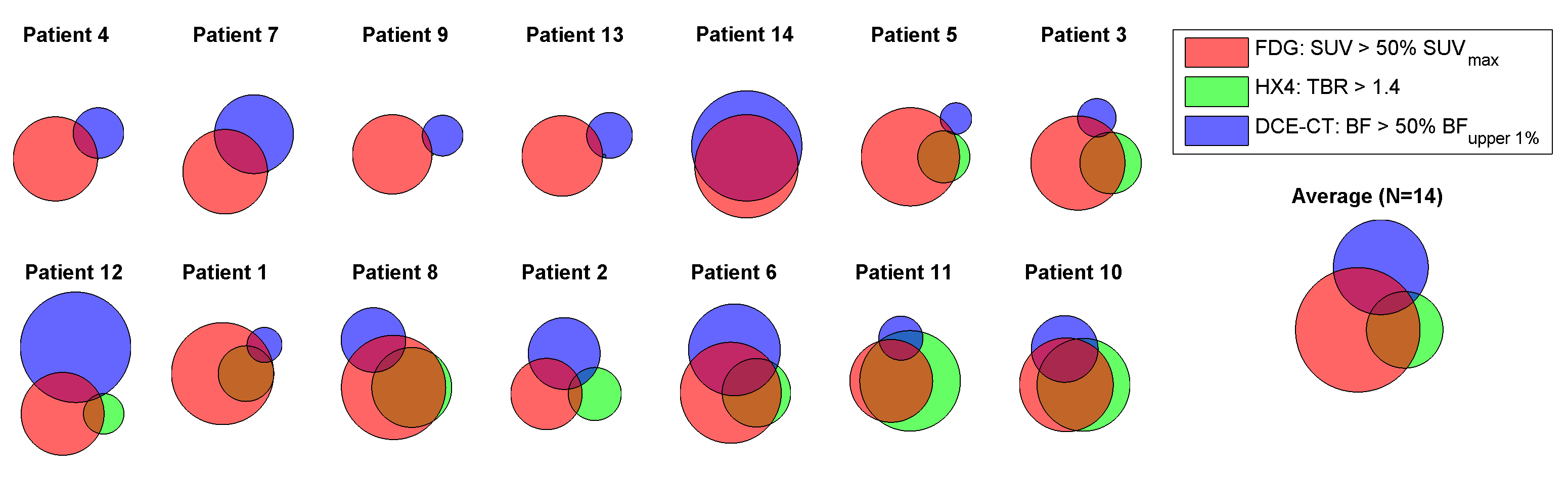


Suppl. Figure 1: Venn diagrams showing schematically the overlap between the high metabolic regions (FDG, red), hypoxic regions above a TBR of 1.4 (HX4, green) and increased perfusion blood flow regions (DCE-CT, blue) per patient. The patients are ordered according to the overlap between hypoxia and blood flow. The right diagram shows the average overlap volumes for all patients. Note that the five patients do not have an hypoxic volume according to the TBR > 1.4 definition.
