# Supplementary material for: Multiparametric imaging of patient and tumour heterogeneity in non-small-cell lung cancer: quantification of tumour hypoxia, metabolism and perfusion
Source: Eur J Nucl Med Mol Imaging. 2015 Sep 4;43:240–8. doi: 10.1007/s00259-015-3169-4 (PMC4700090; doi:10.1007/s00259-015-3169-4)
Supplement: Supplementary file 2 — (DOCX 22 kb) [file 259_2015_3169_MOESM2_ESM.docx]

Suppl. Table 1: Overview of the correlation coefficients (Spearman) on a population level (N=14). Lower triangle are the correlation coefficients, the top triangle represent the respective p-values (significant values < 0.05 indicated in bold). For calculating the correlation coefficients, the various individual parameters (except for hypoxic volume and fraction) were averaged over the primary tumour representing the average value of the entire volume.

|  | p-value  Correlation coefficient | **Hypoxia PET** | | | **FDG-PET** | **DCE-CT** | |
| --- | --- | --- | --- | --- | --- | --- | --- |
|  |  | **Hypoxic Volume** | **Hypoxic Fraction** | **Hypoxia (TBR)** | **Mean SUV** | **Blood Flow** | **Blood Volume** |
| **Hypoxia PET** | **Hypoxic Volume (TBR>1.4)** |  | **<0.001** | **0.008** | 0.873 | **0.007** | **0.018** |
|  | **Hypoxic Fraction (TBR>1.4)** | **0.894** |  | **<0.001** | 0.837 | **0.029** | 0.130 |
|  | **Hypoxia Tumour-to-background (TBR)** | **0.672** | **0.897** |  | 0.584 | **0.039** | 0.513 |
| **FDG-PET** | **Mean SUV** | 0.047 | 0.061 | 0.160 |  | 0.681 | 0.670 |
| **DCE-CT** | **Blood Flow** | **-0.681** | **-0.582** | **-0.556** | -0.121 |  | **0.026** |
|  | **Blood Volume** | **-0.618** | -0.425 | -0.191 | -0.125 | **0.591** |  |
